# Caveolin-1 Alleviates Acetaminophen-Induced Fat Accumulation in Non-Alcoholic Fatty Liver Disease by Enhancing Hepatic Antioxidant Ability *via* Activating AMPK Pathway

**DOI:** 10.3389/fphar.2021.717276

**Published:** 2021-07-07

**Authors:** Jiarong Wang, Wei Jiang, Jiao Xin, Weiju Xue, Congjian Shi, Jiagen Wen, Yan Huang, Chengmu Hu

**Affiliations:** ^1^ Inflammation and Immune Mediated Diseases Laboratory of Anhui Province, Hefei, China; ^2^ School of Pharmacy, Institute for Liver Diseases of Anhui Medical University, Anhui Medical University, Hefei, China; ^3^ Anhui Institute of Innovative Drugs, Key Laboratory of Anti-inflammatory and Immune Medicine, Ministry of Education, Hefei, China

**Keywords:** caveolin-1, non-alcoholic fatty liver disease, oxidative stress, acetaminophen, adenosine monophosphate-activated protein kinase

## Abstract

Non-alcoholic fatty liver disease (NAFLD) is an independent risk factor for acute liver injury caused by overuse of acetaminophen (APAP). Caveolin-1 (CAV1), a regulator of hepatic energy metabolism and oxidative stress, was found to have a protective effect against NAFLD in our previous study. However, it remains unclear whether CAV1 has a protective effect against APAP-induced hepatotoxicity in NAFLD. The aim of this study was to determine whether CAV1 inhibits oxidative stress through the AMPK/Nrf2/HO-1 pathway to protect the liver from fat accumulation exacerbated by APAP in NAFLD. In this study, seven-week-old C57BL/6 male mice (18–20 g) were raised under similar conditions for *in vivo* experiment. *In vitro*, L02 cells were treated with A/O (alcohol and oleic acid mixture) for 48 h, and APAP was added at 24 h for further incubation. The results showed that the protein expression of the AMPK/Nrf2 pathway was enhanced after CAV1 upregulation. The effects of CAV1 on fat accumulation, ROS, and the AMPK/Nrf2 anti-oxidative pathway were reduced after the application of CAV1-siRNA. Finally, treatment with compound C (an AMPK inhibitor) prevented CAV1 plasmid-mediated alleviation of oxidative stress and fat accumulation and reduced the protein level of Nrf2 in the nucleus, demonstrating that the AMPK/Nrf2/HO-1 pathway was involved in the protective effect of CAV1. These results indicate that CAV1 exerted a protective effect against APAP-aggravated lipid deposition and hepatic injury in NAFLD by inhibiting oxidative stress. Therefore, the upregulation of CAV1 might have clinical benefits in reducing APAP-aggravated hepatotoxicity in NAFLD.

## Introduction

Acetaminophen (APAP) is one of the most commonly used antipyretic and analgesic over-the-counter (OTC) drugs worldwide. Nevertheless, its long-term use or overdose of APAP is the most common cause of acute liver failure and intentional or accidental death in the northern hemisphere (Lee, 2017). Notably, long-term APAP ingestion has been reported to aggravate chronic liver injury in some patients, including NAFLD (Michaut et al., 2014).

Non-alcoholic fatty liver disease (NAFLD) is a chronic liver disease with an increasing prevalence worldwide and is characterized by liver triacylglycerol build-up (Tanaka et al., 2019). Clinical and experimental evidence also demonstrates that excessive APAP consumption in NAFLD aggravates risk and severity of hepatic injury (Michaut et al., 2014; Arconzo et al., 2021). Although the effect of steatosis on drug toxicity remains controversial, it is worth noting that a recent study showed that a high-fat diet (HFD) can induce the expression of drug-metabolizing enzymes (including CYP2E1) in the liver of C57BL/6 male mice (Wang et al., 2020), which may be responsible for the vulnerability of NAFLD to APAP. Moreover, in our previous study, APAP exacerbated lipid deposition in NAFLD by inhibiting autophagy *via* the AMPK/mTOR pathway (Shi et al., 2019). Importantly, most of the overweight and obese people develop fatty liver disease, but many patients do not exhibit symptoms, so they are highly likely to overuse APAP unknowingly (Tittarelli et al., 2017). So far, N-acetylcysteine (NAC) is the only clinical antidote against hepatotoxicity following APAP overdose, but it is only effective when administered within 8–10 h of APAP toxicity, and may cause some side effects such as nausea and vomiting (Fisher and Curry, 2019). Thus, more specific pharmacological treatment need be fully explored and established.

Caveolin-1 (CAV1), a principal scaffolding protein of caveolae, plays an important role in multiple biological processes such as cell signal transduction, substance transmembrane transport, glucose, and lipid metabolism (Fernandez-Rojo and Ramm, 2016). Studies have demonstrated that CAV1 is an important regulator of liver function, hepatic energy metabolism, and oxidative stress (Martinez-Outschoorn et al., 2015; Wang et al., 2017). The amino acid sequence 82–101 of CAV1 is called the caveolin-1 scaffolding domain (Caveolin-1 scaffolding domain, CSD), which is involved in interacting with signaling-related proteins to modulate different signaling cascades (Vogel et al., 2019; Xue et al., 2020). When connected with antennapedia homeodomain (AP), CSD can quickly enter cells and tissues. It has been confirmed that CSD could inhibit the progression of liver fibrosis by restoring the effect of CAV1 (Lu et al., 2018). However, the role of CAV1 in the liver is controversial in different experimental models. CAV1 knockout was reported to augment hepatic steatosis in the livers of high fat fed mice with NAFLD (Li et al., 2017). Our previous study also indicated that CAV1 alleviates lipid deposition in NAFLD by promoting autophagy (Xue et al., 2020), while another study presented contradictory findings that a significant attenuation of APAP-induced liver injury in the CAV1 KO animals (Gardner et al., 2010). However, there have been no studies to explore the role of CAV1 in the hepatotoxicity of APAP based on NAFLD.

Recently, oxidative stress is considered to play a pivotal role in the development of NAFLD, which can result in liver cell damage, excessive fatty acids in hepatocytes, and subsequent energy depletion (Borrelli et al., 2018; Masarone et al., 2018). Further, oxidative stress is also a key pathogenesis of APAP-induced hepatotoxicity (Du et al., 2016). N-acetyl-p-benzoquinone imine (NAPQI), a toxic metabolite produced by APAP through the cytochrome P450 system, can be detoxified rapidly by conjugation with glutathione (GSH). However, when APAP is overused or used in sensitive individuals, excessive NAPQI can deplete GSH and bind covalently to the cysteinyl residues to form toxic APAP adducts (APAP-AD) (Yan et al., 2018). Interestingly, it was reported that a significant attenuation of APAP-induced hepatotoxicity in the CAV1 KO animals is not related to alterations in the antioxidant defense. In contrast, the protective effect of CAV1 on alcoholic fatty liver was reported to lie in reducing oxidative stress (Gao et al., 2014; Lee et al., 2017). Hence, the exact effect of CAV1 on liver injury induced by APAP in NAFLD and its molecular mechanism still need to be investigated.

AMP-activated protein kinase (AMPK) is a serine/threonine kinase that can be activated by phosphorylation on Thr172 in the activation loop of the catalytic (*α*) subunit by an upstream kinase to regulate energy homeostasis and metabolic stress (Herzig and Shaw, 2018). Investigations have suggested that CAV1 is associated with the activation of AMPK (Salani et al., 2012; Martinez-Outschoorn et al., 2015; Baek et al., 2017). Moreover, previous studies revealed that metformin induces AMPK phosphorylation depending on the expression of CAV1 (Salani et al., 2012; Nwosu et al., 2016). Inhibition of CAV1 expression induced a decrease in AMPK phosphorylation and AMP/ATP ratio (Das et al., 2012; Yamamoto et al., 2018). Moreover, AMPK-mediated Nrf2/HO-1 signaling pathway can attenuate NAFLD and APAP-induced hepatotoxicity by reducing oxidative stress (Wang et al., 2019; Xie et al., 2020).

Accordingly, the aim of this study was to explore the exact role of CAV1 in liver injury exacerbated by APAP in NAFLD and whether its potential mechanisms were associated with inhibiting oxidative stress through the AMPK/Nrf2/HO-1 pathway. Therefore, in order to simulate liver damage caused by APAP in NAFLD, we used a high-fat diet animal model that is similar to the pathological manifestation of early stage NAFLD. Given the effects of different doses of APAP on liver injury and mortality in mice, the optimal dose of APAP (100 mg/kg) in our previous study was used in the current experiment. Besides, in order to ensured better model stability, we chose the alcohol and oleic acid mixture and L02 cells according to previous studies to establish steatosis model *in vitro*.

## Materials and Methods

### Animal Experiments

Seven-week-old C57BL/6 male mice (18–20 g) were obtained from the Laboratory Animal Center of Anhui Medical University. All animals were raised under standard conditions (24 ± 2°C, 12-h day-night cycle). Mice were allowed access to feed and water during the study period. The study was conducted in accordance with the requirements of the Animal Care and Ethics Committees of Anhui Medical University (NO: LLSC20190279). Mice were randomly divided into seven groups (*n* = 8 per group): 1) normal control group (NC), 2) high-fat diet group (HFD), 3) HFD with DMSO group (HFD + DMSO), 4) HFD with APAP (100 mg/kg) (Solarbio, Beijing, China) (HFD + APAP), 5) HFD with CSD (4 mg/kg, dissolved in 0.5% DMSO, Sangon Biotech, Shanghai, China) (HFD + CSD), 6) HFD with APAP and CSD (HFD + APAP + CSD), and 7) HFD with APAP and NAC (200 mg/kg, positive control, Sigma, United States) (HFD + APAP + NAC). The NC group was fed a normal diet (20% calories from protein, 10% calories from fat, 70% calories from carbohydrate; 3.5 kcal/g diet; TP26312; TROPHIC, Nantong, China), whereas the other groups were fed a high-fat diet (HFD) (15% calories from protein, 42% calories from fat, 43% calories from carbohydrate, 4.5 kcal/g diet; TP26300; TROPHIC, Nantong, China).

The experimental period lasted for 56 days. On the 42nd day of the study, mice in the designated group received either an intraperitoneal injection of CSD (4 mg/kg/d) or an equal volume of 0.5% DMSO as vehicle control for 2 weeks. Desalted CSD (amino acids 82–101 of CAV1; DGIWKASFTTFTVTKYWFYR) powder was synthesized and analyzed to confirm its purity was more than 97% by Sangon Biotech Corporation (Shanghai, China). Desiccated peptides were weighed, dissolved in DMSO to 10 mM, and diluted to 2.5 mM with freshly prepared distilled water. On the last day of the study, mice were administered APAP (100 mg/kg) or an equal volume of PBS by gavage (only once), whereas NAC group mice were intraperitoneally injected with NAC (200 mg/kg, Beyotime, China) an hour before APAP administration. The fresh APAP solution was prepared by dissolving it in phosphate-buffered saline (PBS). After fasting for 24 h, blood and liver tissue samples were collected for blood biochemical and histopathological analyses, respectively.

### Biochemical Measurements

Serum alanine aminotransferase (ALT) and aspartate aminotransferase (AST), serum triglyceride (TG), glutathione (GSH), and lipid peroxidation malondialdehyde (MDA) were measured using relative assay kits (Jiancheng, Nanjing, China) with a microplate reader (Biotek, United States).

### Hepatic Biochemical Analysis

Portions of liver tissues were homogenized and dissolved in PBS to measure the activity of superoxide dismutase (SOD) using the Total SOD Assay Kit with WST-8 (Beyotime, China) according to the manufacturer’s instructions.

### Histopathologic Examination of Liver Tissue

Portions of mouse livers were stored in 4% paraformaldehyde, fixed for 48 h, embedded in paraffin, cut into thin slices, and stained with hematoxylin and eosin (H and E). Oil Red O staining was conducted on 5-µm frozen liver sections to observe lipid droplet deposition in liver tissue.

### Detection of ROS Production in the Liver

The cell-permeable fluorophore DHE was used to evaluate in the production of ROS. DHE is oxidized by ROS to a novel product that binds to DNA, enhancing intracellular fluorescence. The images were captured using a fluorescence microscope at 400× magnification (excitation at 490 nm and emission at 610 nm). The fluorescence intensity values were quantified using Image J 1.48u software (National Institutes of Health, United States).

### Cell Culture and Treatment

Human normal hepatocytes L02 cells were obtained from the Cell Bank of the Chinese Academy of Sciences (Shanghai, China). Cells were grown and maintained in Dulbecco’s modified Eagle’s medium (DMEM, Gibco, United States) supplemented with 10% fetal bovine serum (FBS, Every Green, China) and 1% penicillin/streptomycin at a constant temperature of 37°C in a 5% CO2 incubator.

To evaluate the effect of APAP on steatosis in L02 cells, a model of hepatocyte steatosis *in vitro* was established by co-treating L02 cells with 87 mM alcohol and 100 µM oleic acid (A/O) for 48 h. During the last 24 h of the 48-h treatment, different concentrations of APAP were added, and the plate was incubated for another 24 h. The cells were divided into five groups: 1) normal group (N), 2) alcohol and oleic acid group (A/O), 3) A/O with 2 mM APAP, 4) A/O with 4 mM APAP, and 5) A/O with 8 mM APAP.

### Oil Red O Staining and Measurement of Intracellular TG

The cells were seeded into 6-well plates and cultured with designated reagents and drugs, fixed with 4% paraformaldehyde, and stained with a freshly prepared working solution of Oil Red O at room temperature. Thirty minutes later, stains were removed, and the background stains were washed until they were negligible. After 2 h, orange-red lipid droplets were observed in the cells under the bright field of fluorescence inverted microscope (Olympus, Tokyo, Japan). The activity of TG in cells were measured using analysis kits (Jiancheng, Nanjing, China) and calculated according to the manufacturer’s instructions.

### Cell Transfection and Compound C Administration

Overexpression of CAV1 was achieved using the GV146-CAV1 plasmid acquired from GeneChem Corporation (Shanghai, China). L02 cells were transfected with GV146-CAV1 or GV146-Control using Lipofectamine3000 (Polyplus-jetPRIME, Illkirch, France) as recommended by the manufacturer’s protocol. After transfection for 6 h, the medium was replaced with a fresh medium, and the cells were further treated with A/O and APAP. To determine whether AMPK was involved in CAV1-mediated liver protection, 2.5 µM AMPK inhibitor compound C (Medchem Express, United States) was administered 1 h prior to A/O treatment. L02 cells were collected for western blotting, ROS determination, and Oil Red O staining.

Small interfering RNA (siRNA) for CAV1 and negative control-siRNA were synthesized by GenePharma Corporation. The sequences were as follows: CAV1-siRNA, 5′-CCG​CAU​CAA​CUU​GCA​GAA​ATT-3′ and 5′-UUU​CUG​CAA​GUU​GAU​GCG​GTT-3′; and Control-siRNA, 5′-UUC​UCC​GAA​CGU​GUC​ACG​UTT-3′ and 5′-ACG​UGA​CAC​GUU​CGG​AGA​ATT-3′.

L02 cells were transfected with CAV1-siRNA and Control-siRNA for 6 h using Lipofectamine 3,000 reagent and induced as above described. Finally, the cells were harvested for the follow-up experiments.

### Detection of Intracellular ROS Production

After L02 cells were treated with the fluorescence probe DCFH-DA (Bestbio, Shanghai, China) according to the manufacturer’s instructions, the fluorescence intensities were measured by flow cytometry (Beckman Coulter Epics XL, United States) at excitation and emission wavelengths of 488 and 525 nm, respectively.

### Western Blot Analysis

The total proteins of liver tissues and L02 cells were extracted using RIPA lysis buffer containing 1% protease and phosphatase inhibitors (Solarbio, Beijing, China). The lysates were separated using sodium dodecyl sulfate-polyacrylamide gel electrophoresis (SDS-PAGE) after the protein concentration was determined using a BCA protein assay kit (Beyotime, China). Subsequently, the proteins were transferred onto polyvinylidene difluoride (PVDF) membranes (Millipore Corp, Billerica, MA), which were then blocked with 5% non-fat milk and incubated with primary antibodies at 4°C overnight. After the membranes had been washed with TBST, they were incubated with peroxidase-labeled secondary antibody (1:5,000, Zhongshan Jinqiao, China) at 37°C for 1 h. Protein blots were detected using an enhanced chemiluminescence kit (ECL-plus, Thermo Scientific) and quantified using Image J Software. The primary antibodies used in this study were: CAV1, SREBP-1c, HO-1, AMPK (Abcam, Cambridge, United States), p-AMPK (Thr172), β-actin (Bioss, Beijing, China), and Nrf2, Lamin-B (ProteinTech Group, Chicago, United States).

### Preparation of Cytoplasmic and Nuclear Protein Extracts

Cytoplasmic and nuclear extracts in liver tissues and cells were prepared using a nuclear and cytoplasmic extraction kit (BestBio, Shanghai, China) according to the manufacturer’s instructions. The quantitative changes of Nrf2 in the nucleus or cytoplasm were detected by western blotting. Lamin-B1 was used as a nuclear envelope marker. β-actin was used as a cytosolic protein marker.

### Statistical Analysis

All experimental data were analyzed using GraphPad and SPSS (version 18.0). The data were expressed as the (mean S.D.). Comparisons between multiple groups were made by one-way analysis of variance (ANOVA) followed by Duncan s test. Differences were considered significant if the P value was less than 0.05.

## Results

### CSD Reduced Lipid Deposition and Liver Injury Caused by APAP in NAFLD

**FIGURE 1:**
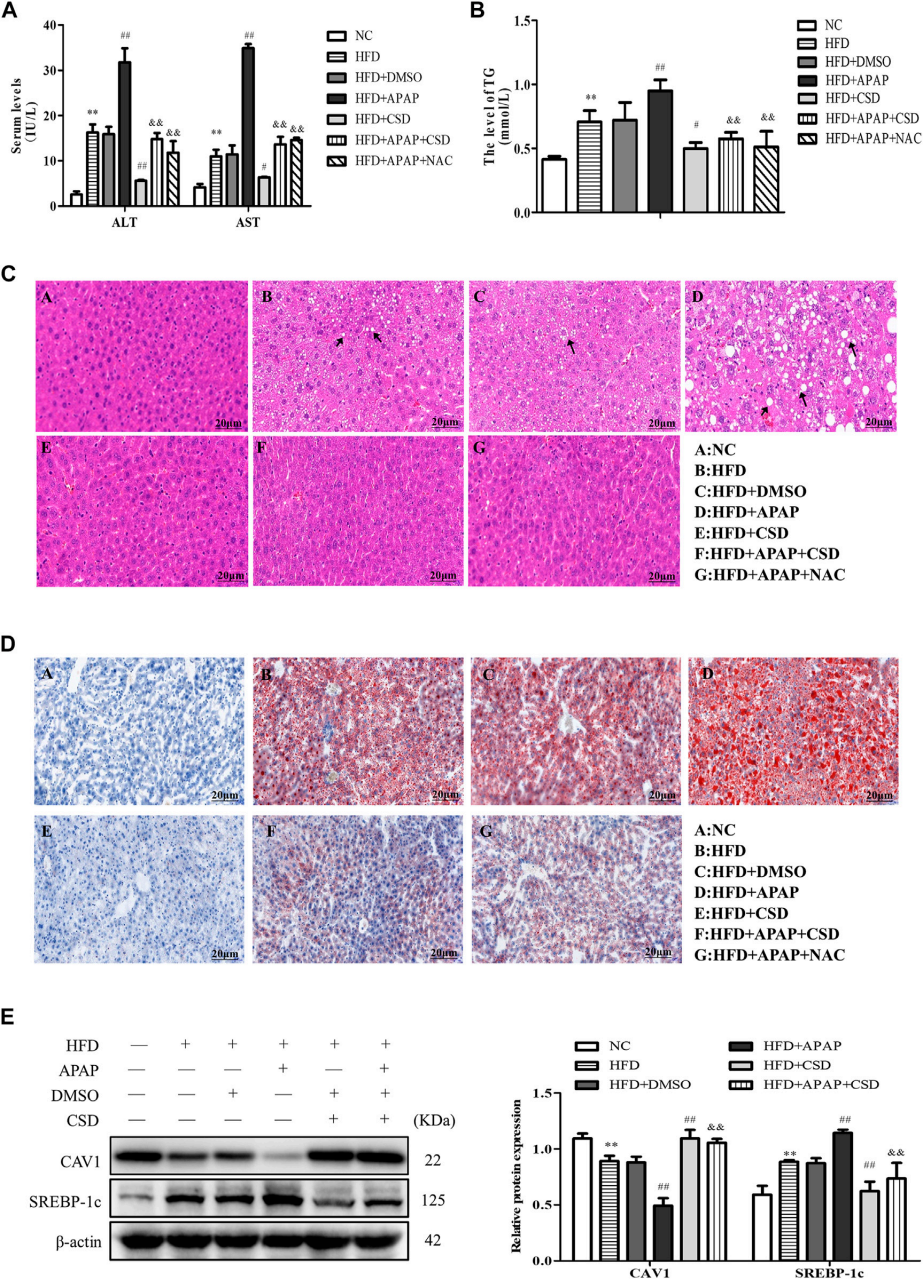
CSD reduced lipid deposition and liver injury caused by APAP. Male C57BL/6J mice were fed an HFD (high-fat diet), while APAP (100 mg/kg) was given by gavage on the last day. N-Acetylcysteine (NAC) was tested as a positive control. **(A)** The serum activity of ALT and AST and **(B)** TG. **(C)** Representative images of hematoxylin and eosin staining results for liver tissue sections. **(D)** Oil Red O staining of lipid droplets in liver. Data represent mean ± S.D. of each group. (*n* = 8). **p < 0.01 vs. NC group; ^#^ *p* < 0.05 and ^##^ *p* < 0.01 vs. HFD group; ^&&^ *p* < 0.01 vs. HFD + APAP group. **(E)** Western blot analysis of the relative protein levels of CAV1 and SREBP-1c were measured in liver tissue. These bands are from separate membranes. Data represent mean ± S.D. of each group. (*n* = 3). **p < 0.01 vs. NC group; ^##^ *p* < 0.01 vs. HFD group; ^&&^ *p* < 0.01 vs. HFD + APAP group.

The activity of ALT, AST, and TG in the HFD group were significantly higher than those in the NC group. Compared to those in the HFD group, the above parameters were significantly higher in the HFD + APAP group. However, CSD treatment reduced the levels of these parameters in the HFD + APAP group (Figures 1A,B). Moreover, the HFD + APAP group exhibited more severe intrahepatic necrosis and extensive red lipid droplets than the HFD group, but treatment with CSD reduced the severity of these phenomena (Figures 1C,D). The level of SREBP-1c was significantly increased, whereas that of CAV1 protein expression was significantly decreased in the HFD group. These changes were more significant in the HFD + APAP group than in the HFD group (Figure 1E). These results indicated that CSD effectively reduced APAP-aggravated hepatic injury and lipid deposition in NAFLD.

### CSD Attenuated Oxidative Stress Caused by APAP in NAFLD

**FIGURE 2:**
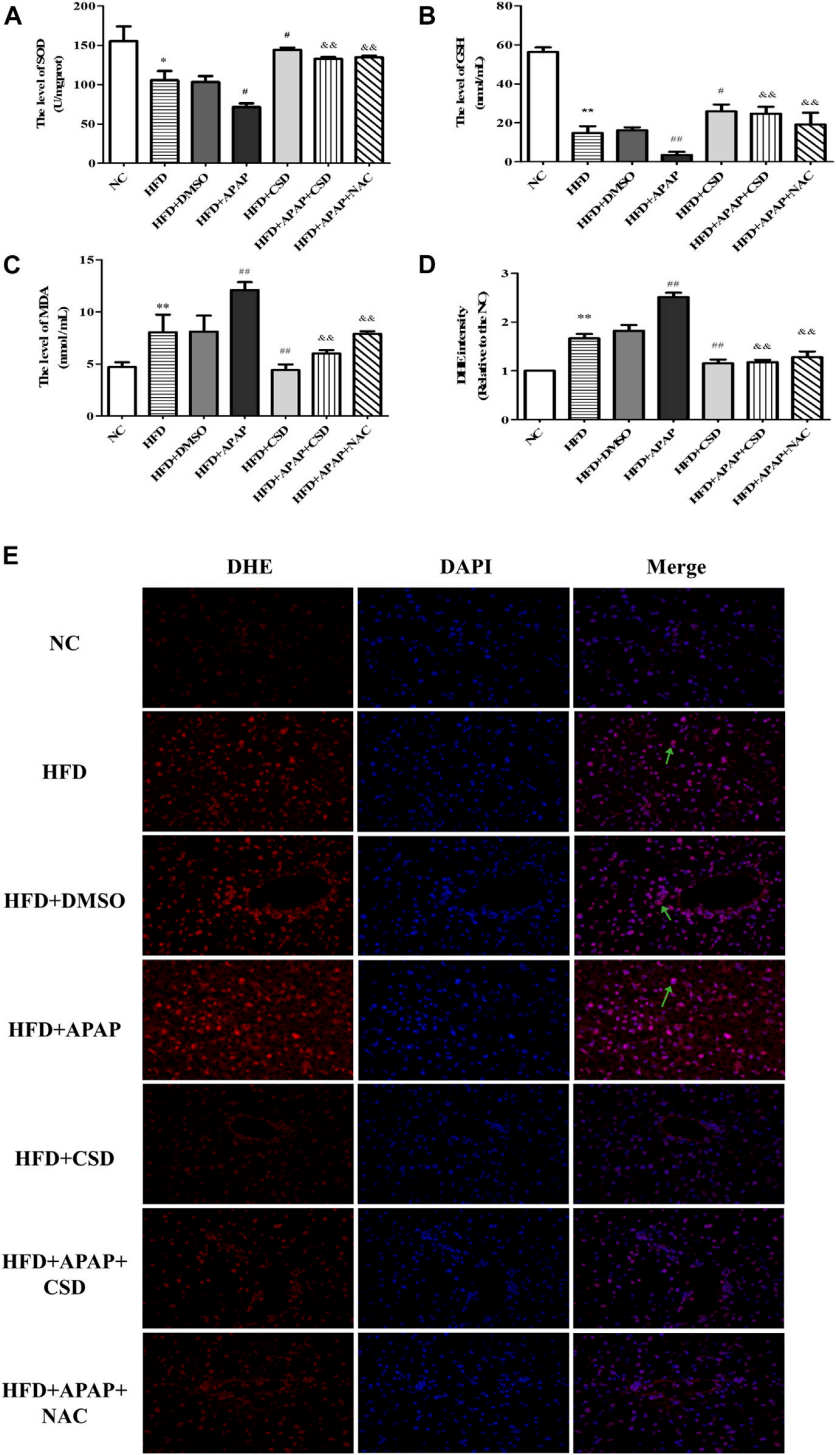
CSD attenuated oxidative stress caused by APAP. **(A,B)** liver superoxide dismutase (SOD) activity and Serum glutathione (GSH). **(C)** malondialdehyde (MDA) level. Data represent mean ± S.D. of each group. (*n* = 8). *p < 0.05 and **p < 0.01 vs. NC group; ^#^ *p* < 0.05 and ^##^ *p* < 0.01 vs. HFD group; ^&&^ *p* < 0.01 vs HFD + APAP group. **(D,E)** Reactive oxygen species (ROS) production was assessed by DHE staining. DAPI was used for nuclear staining (blue). Red color represents ROS staining (*n* = 3 independent experiments). Data represent mean ± S.D. of each group. (*n* = 3). **p < 0.01 vs. NC group; ^##^ *p* < 0.01 vs. HFD group; ^&&^ *p* < 0.01 vs. HFD + APAP group.

The biochemical measurements revealed that the activity of SOD and GSH were lower and those of MDA were significantly higher in the HFD group than in the NC group. These parameters changed significantly in the HFD + APAP group when compared to the HFD group. Nevertheless, CSD treatment reversed these changes (Figures 2A–C). In addition, the ROS fluorescence intensity was highest in the HFD + APAP group, followed by the HFD group and the NC group. However, the ROS fluorescence intensities were significantly reduced by HFD + CSD treatment (Figures 2D,E). The above results confirmed the inhibitory effect of CSD on oxidative stress aggravated by APAP in NAFLD.

### Effect of CSD on the Body Weight and Liver Index of Mice in Each Group

**FIGURE 3:**
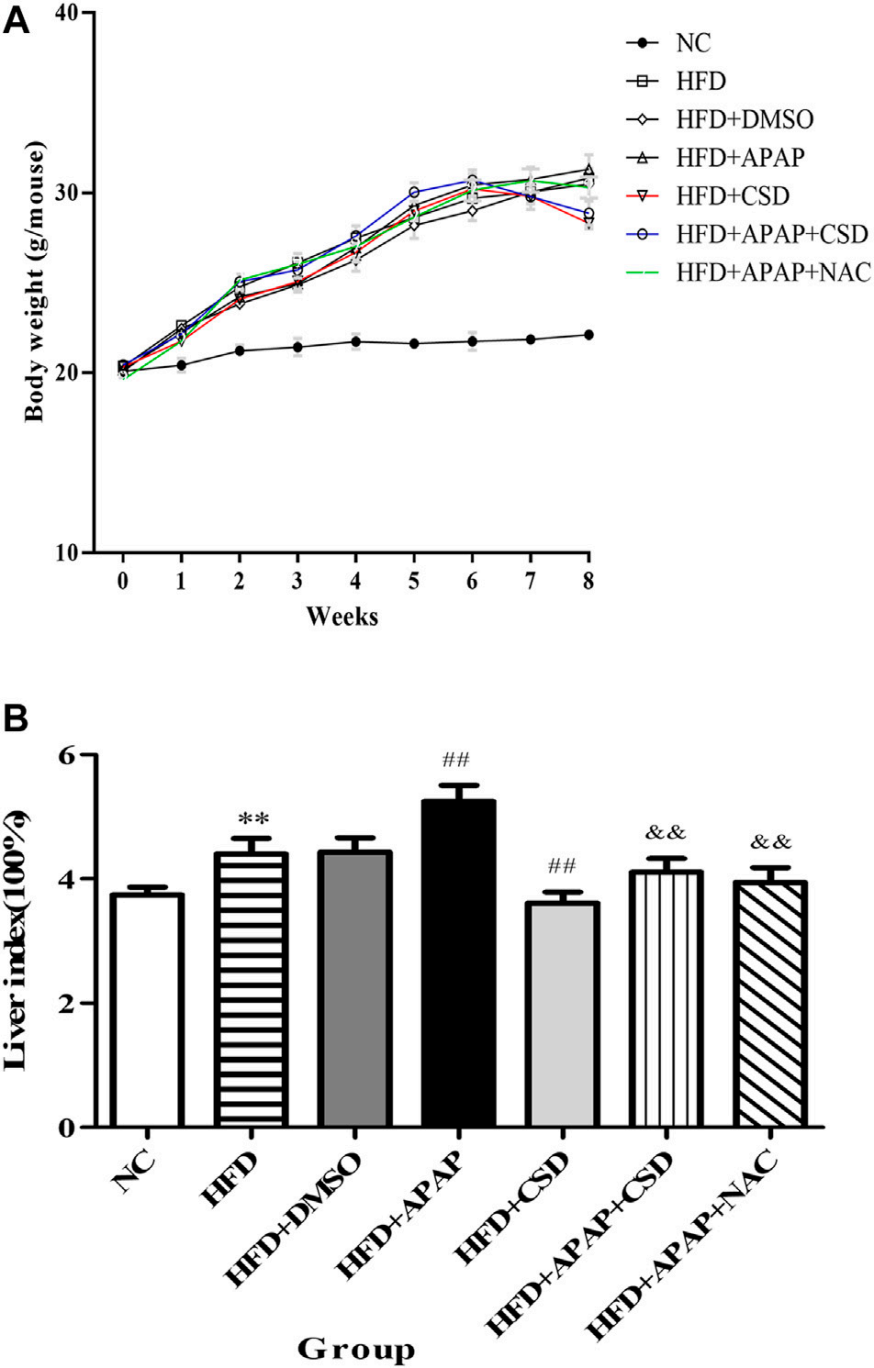
Effect of CSD on the body weight and liver index of mice in each group. **(A)** The body weight of mice in each group. **(B)** The liver index of mice in each group. Data represent mean ± S.D. of each group. (*n* = 8). **p < 0.01 vs. NC group; ^##^ *p* < 0.01 vs. HFD group; ^&&^ *p* < 0.01 vs. HFD + APAP group.

The body weight of the mice was counted after randomized grouping. After 1 week of modeling period, there were significant differences in body weight between the NC group, the HFD group and all APAP-treated mice. With the increase of modeling time, the body weight of the HFD group and all APAP-treated mice was significantly higher than that of the NC group, while the final body weight of the APAP-treated group was higher than that of the HFD group. After CSD treatment, the mice began to lose weight gradually (Figure 3A). Liver index is one of the important indexes reflecting the degree of liver injury and fat accumulation in liver. As shown in the figure, liver index of HFD group and all APAP-treated mice was significantly higher than that of NC group. The liver index was significantly reduced after CSD treatment (Figure 3B).

### Expression Level of CAV1 and p-AMPK, Lipid Accumulation, and Oxidative Stress in L02 Cells Induced by A/O and APAP

**FIGURE 4:**
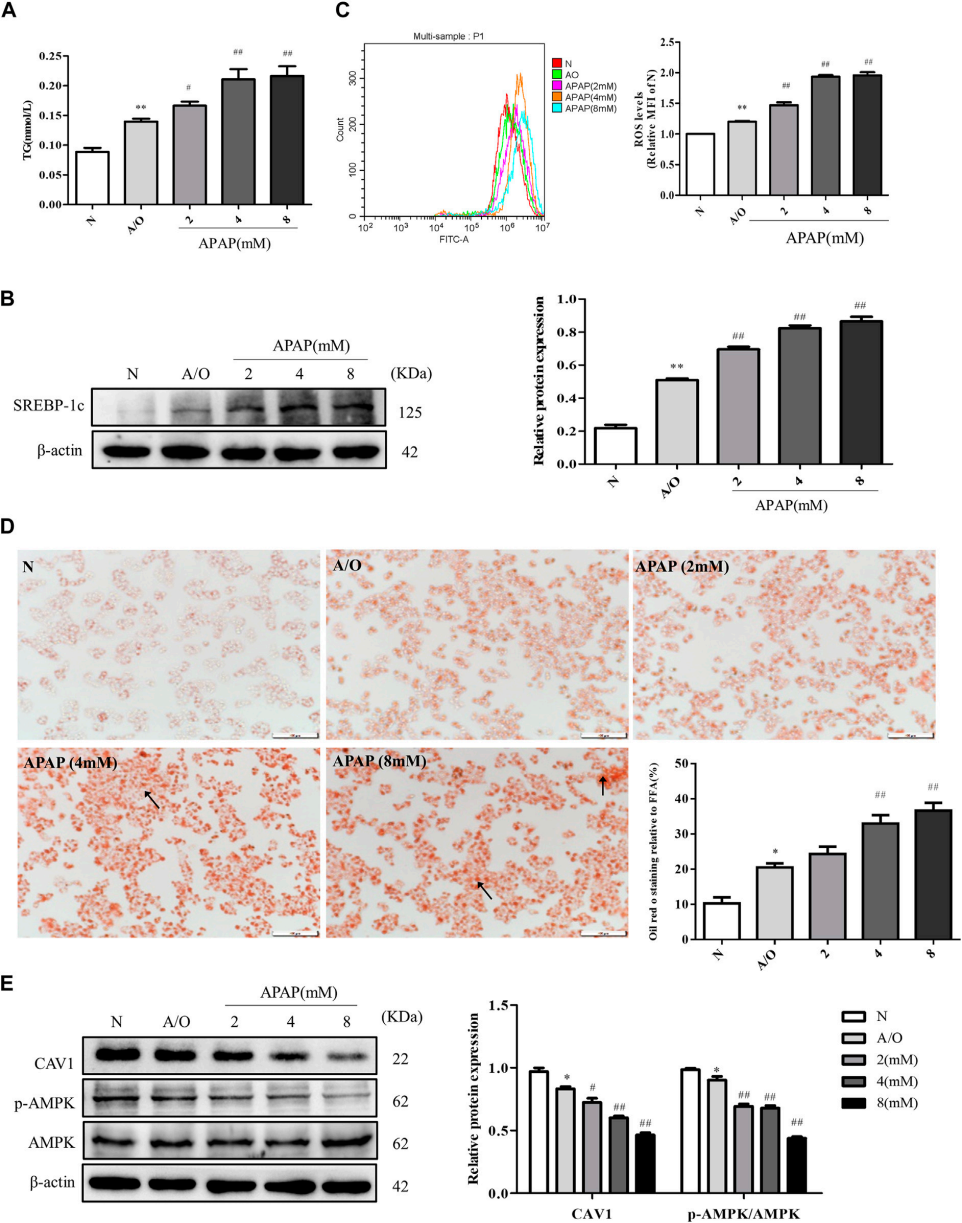
APAP exacerbated lipid accumulation and oxidative stress in L02 cells induced by A/O. **(A)** The TG levels in L02 cells treated with APAP and A/O. **(B)** Western blot analysis of SREBP-1c protein level was measured. **(C)** The analysis of ROS production by flow cytometry. **(D)** Oil red O staining of lipid droplets in L02 cells treated with different dose of APAP and A/O. **(E)** The effects of different dose of APAP on CAV1 protein level and p-AMPK protein level were measured by Western blot. These bands are from separate membranes. Data represent mean ± S.D. of each group. (*n* = 3). *p < 0.05 and **p < 0.01 vs. N group; ^#^ *p* < 0.05 and ^##^ *p* < 0.01 vs. A/O group.

The levels of TG, SREBP-1c protein expression, and ROS were significantly increased in A/O-treated cells and were further increased after APAP treatment (Figures 4A–C). Likewise, Oil Red O staining showed prominent orange lipid droplets in A/O-treated cells and were further increased after APAP administration (Figure 4D). Furthermore, western blotting revealed that the protein levels of CAV1 and p-AMPK were significantly reduced in A/O-treated cells and further decreased after APAP treatment (Figure 4E). These results were consistent with those *in vivo*, suggesting that the CAV1 and AMPK signaling pathways are involved in APAP-induced liver injury.

### CAV1 Alleviated Lipid Accumulation and Oxidative Stress in L02 Cells Induced by A/O and APAP

**FIGURE 5:**
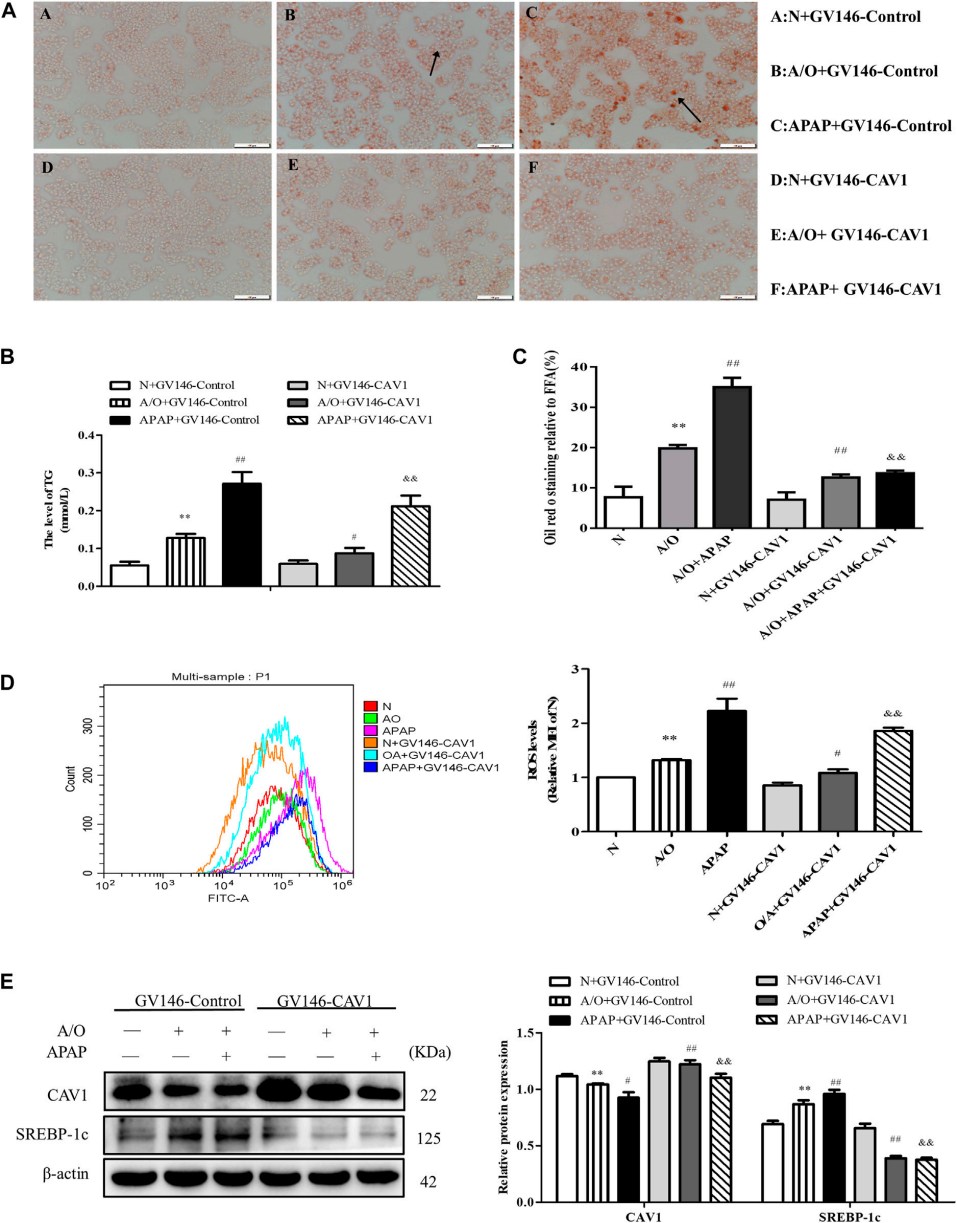
CAV1 alleviated lipid accumulation and oxidative stress in L02 cells induced by A/O and APAP. **(A,C)** Oil Red O staining of lipid droplets in A/O and APAP stimulated cells treated with GV146-CAV1. **(B)** The TG level in A/O and APAP stimulated cells treated with GV146-CAV1. **(D)** ROS levels in A/O and APAP stimulated cells treated with GV146-CAV1. **(E)** Western blot analysis of SREBP-1c and CAV1 protein levels were measured. These bands are from separate membranes. Data represent mean ± S.D. of each group. (*n* = 3). **p < 0.01 vs. Normal + GV146-Control group; ^#^ *p* < 0.05 and ^##^ *p* < 0.01 vs. A/O + GV146-Control group. ^&&^ *p* < 0.01 vs. APAP + GV146-Control group.

We found that upregulation of CAV1 reduced lipid droplet accumulation, TG activity, and ROS content (Figures 5A–D). Western blotting also indicated that the level of SREBP-1c was lower in the GV146-CAV1-treated groups than in the GV146-control-treated groups (Figure 5E).

### CAV1 Silencing Aggravated Lipid Accumulation and Oxidative Stress in L02 Cells Induced by A/O and APAP

**FIGURE 6:**
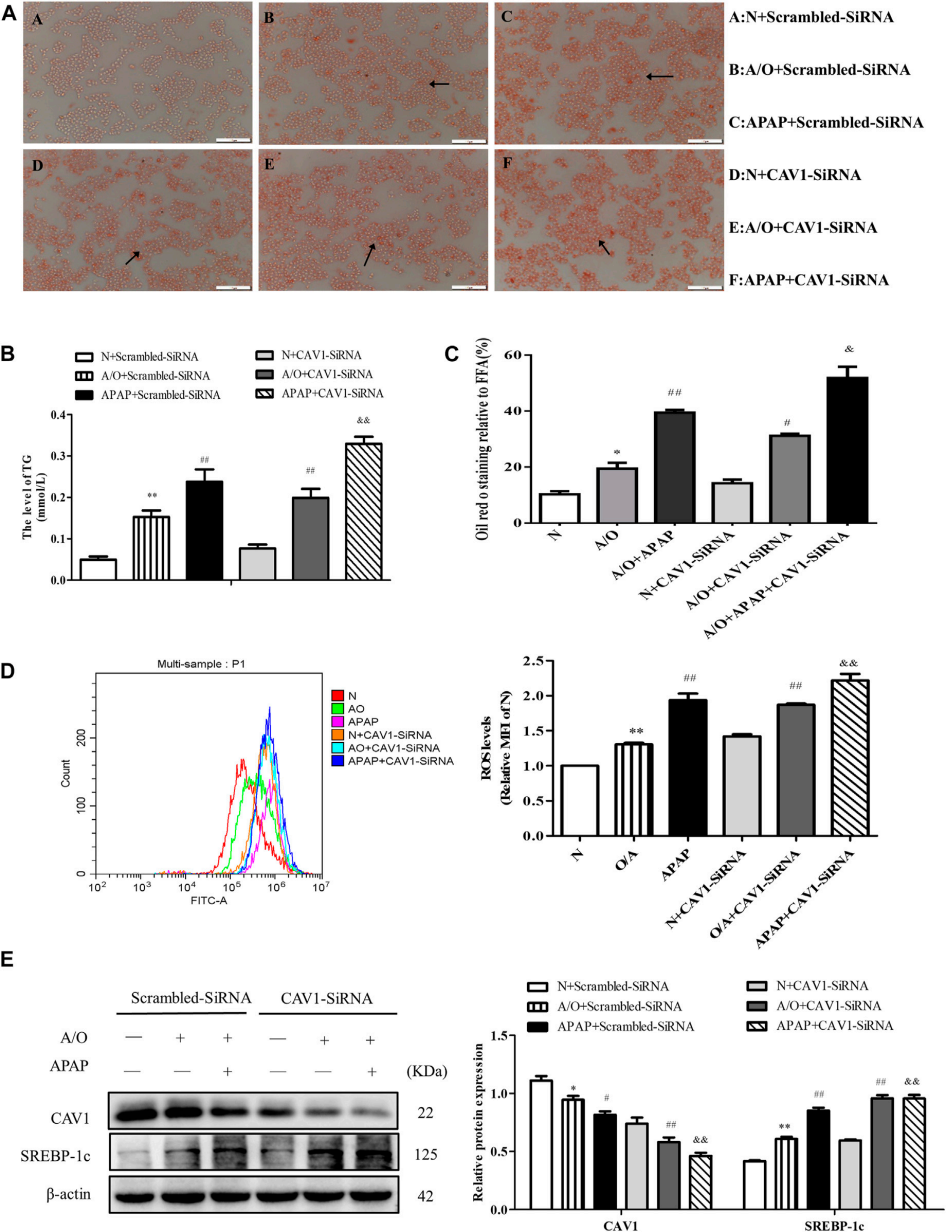
CAV1 silencing exacerbated lipid accumulation and oxidative stress in L02 cells induced by A/O and APAP. **(A,C)** Oil Red O staining of lipid droplets in A/O and APAP stimulated cells treated with CAV1-siRNA. **(B)** The TG level in A/O and APAP stimulated cells treated with CAV1-siRNA. **(D)** ROS levels in A/O and APAP stimulated cells treated with CAV1-siRNA. **(E)** Western blot analysis of SREBP-1c and CAV1 protein levels were measured. These bands are from separate membranes. Data represent mean ± S.D. of each group. (*n* = 3). *p < 0.05 and **p < 0.01 vs. N + Scrambled-SiRNA group; ^#^ *p* < 0.05 and ^##^ *p* < 0.01 vs. A/O+ Scrambled-SiRNA group. ^&&^ *p* < 0.01 vs. APAP + Scrambled-SiRNA group.

CAV1 was downregulated in L02 cells by transfection with CAV1-siRNA. We observed an increase in lipid droplet accumulation, TG activity, and ROS production after the downregulation of CAV1 level (Figure 6A–D). Western blotting revealed that the protein levels of CAV1 were lower whereas the level of SREBP-1c was higher in the corresponding CAV1-siRNA-treated groups than in the control-siRNA-treated groups (Figure 6E).

### CAV1 Increased the Activity of AMPK and the Activation of Nrf2 *in vivo* and *in vitro*

**FIGURE 7:**
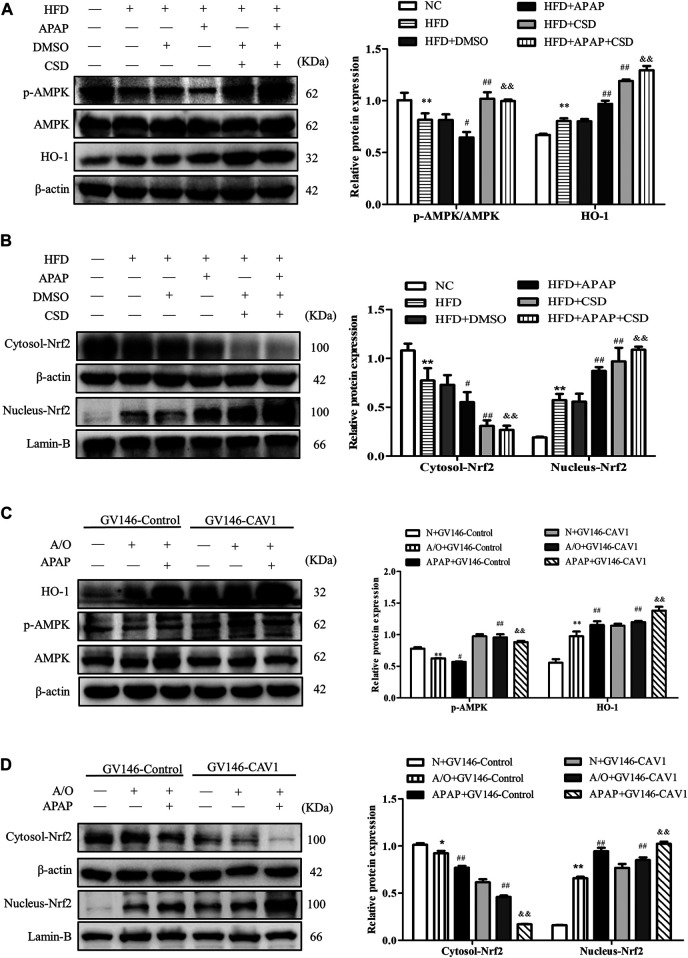
CAV1 increased the level of p-AMPK and the level of Nrf2/HO-1 *in vivo* and *in vitro*. **(A)** Western blot analysis of the ratio of p-AMPK/AMPK and the relative protein level of HO-1 in liver tissues. **(B)** The relative protein levels of Nrf2 in the cytoplasm and nucleus in liver. Data represent mean ± S.D. of each group. (*n* = 8). β-actin and Lamin-B were used as controls for the protein blots. These bands are from separate membranes. *p < 0.05 and **p < 0.01 vs. NC group; ^#^ *p* < 0.05 and ^##^ *p* < 0.01 vs. HFD group; ^&&^ *p* < 0.01 vs. APAP group. **(C)** The ratio of p-AMPK/AMPK and the relative protein level of HO-1 in A/O- and APAP-stimulated cells treated with GV146-CAV1 were measured by Western blot. **(D)** The relative protein levels of Nrf2 in the cytoplasm and nucleus measured in A/O− and APAP-stimulated cells treated with GV146-CAV1. Data represent mean ± S.D. of each group. β-actin and Lamin-B were used as controls for the protein blots. These bands are from separate membranes. Data represent mean ± S.D. of each group. (*n* = 3). **p < 0.01 vs. N + GV146-Control group; ^#^ *p* < 0.05 and ^##^ *p* < 0.01 vs. A/O + GV146-Control group. ^&&^ *p* < 0.01 vs. APAP + GV146-Control group.

p-AMPK expression level was significantly lower in HFD mice than in NC mice. Additionally, p-AMPK had a lower expression level in the HFD + APAP group than in the HFD group; however, CSD treatment resulted in an increase in AMPK phosphorylation, Nrf2 nuclear expression, and HO-1 protein expression in liver tissue and a decrease in Nrf2 level in the cytoplasm (Figures 7A,B). Moreover, the overexpression of CAV1 significantly increased protein levels of p-AMPK and HO-1 (Figure 7C) and promoted Nrf2 nuclear expression to a greater extent when compared with those in the GV146-control-treated groups (Figure 7D).

### CAV1 Silencing Inhibited the Activity of AMPK and the Activation of Nrf2 in L02 Cells Induced by A/O and APAP

**FIGURE 8:**
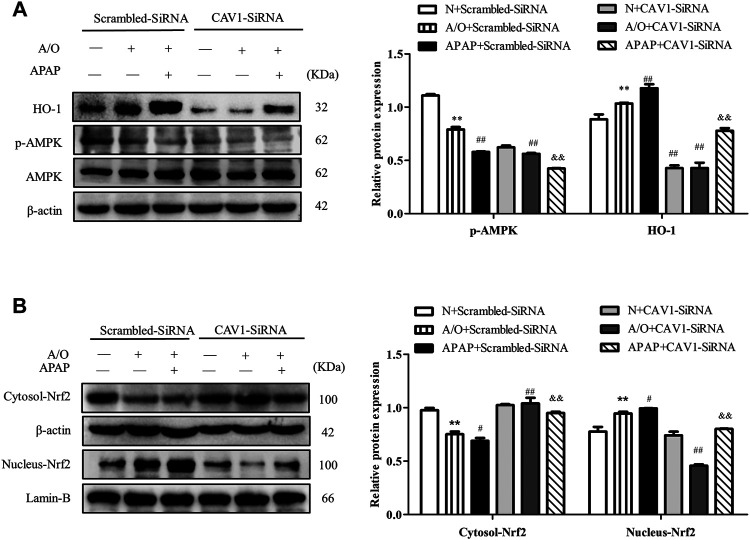
CAV1 silencing inhibited the phosphorylation of AMPK and the level of Nrf2/HO-1 in L02 cells induced by A/O and APAP. **(A)** The ratio of p-AMPK/AMPK and the relative protein level of HO-1 were measured in A/O- and APAP-stimulated cells treated with CAV1-siRNA. **(B)** The relative protein levels of Nrf2 in the cytoplasm and nucleus were measured in A/O- and APAP-stimulated cells treated with GV146-CAV1. These bands are from separate membranes. Data represent mean ± S.D. of each group. (*n* = 3). **p < 0.01 vs. N + Scrambled-SiRNA group; ^#^ *p* < 0.05 and ^##^ *p* < 0.01 vs. A/O + Scrambled-SiRNA group. ^&&^ *p* < 0.01 vs. APAP + Scrambled-SiRNA group.

Consistently, CAV1 silencing decreased the protein levels of p-AMPK and HO-1 (Figure 8A) and reduced the expression of Nrf2 in the nucleus (Figure 8B). These results demonstrate that the protective effect of CAV1 on APAP-induced liver injury in NAFLD may be associated with activation of the AMPK/Nrf2-dependent pathway.

### Inhibition of AMPK Pathway Hampered CAV1-Mediated the Protection

**FIGURE 9:**
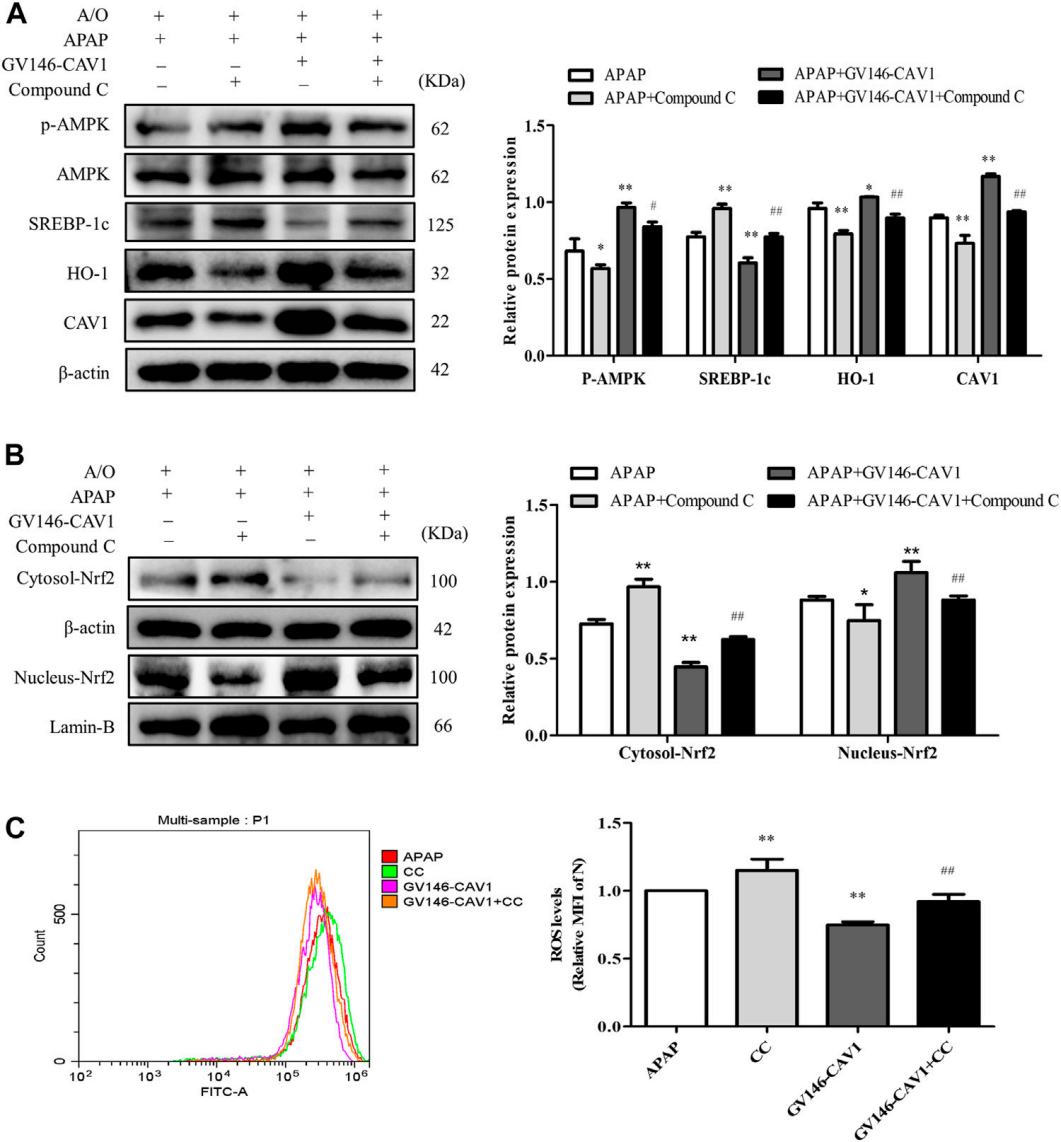
Inhibition of AMPK pathway hampered CAV1-mediated the protection. **(A)** The activation of AMPK, CAV1, SREBP-1c and HO-1 proteins level were analyzed by western blot. **(B)** The relative protein levels of Nrf2 in the cytoplasm and nucleus were measured by western blot. These bands are from separate membranes. **(C)** Intracellular ROS content was analyzed by flow cytometry. Data represent mean ± S.D. of each group. (*n* = 3). *p < 0.05 and **p < 0.01 vs. APAP group; ^#^ *p* < 0.05 and ^##^ *p* < 0.01 vs. APAP + Compound C group.

After compound C treatment, the protein levels of p-AMPK, CAV1, nuclear Nrf2, and HO-1 decreased, whereas the levels of SREBP-1c protein and ROS increased (Figures 9A–C). The treatment of compound C, therefore, reversed the effects of CAV1 overexpression on APAP and A/O L02 cells, suggesting that the liver protective effect of CAV1 in response to APAP toxicity is involved in the regulation of the AMPK/Nrf2 oxidant pathway.

## Discussion

APAP is a widely used OTC drug for the treatment of fever and pain that can be metabolized by the CYP450 system to the toxic compound NAPQI. It is usually detoxified by binding to GSH, but when GSH is depleted, an accumulation of NAPQI occurs, leading to liver damage. It has been reported that the maximum recommended dose can lead to hepatic cytolysis in some individuals, and obesity-related hepatic lesions appear to increase the risk and severity of APAP-induced liver damage (Michaut et al., 2014), but the exact mechanism remains controversial.

NAFLD, as a chronic liver disease, has an increasing incidence (Tanaka et al., 2019). Some studies found that patients with NAFLD have an increased risk of APAP-related mortality (Michaut et al., 2014; Tanaka et al., 2019; Arconzo et al., 2021). Although clinicians should prescribe different dosing regimens for different patients, a large systematic review found that the high incidence of hepatotoxicity of APAP was mostly due to accidental overdose (Hayward et al., 2016). The underlying mechanism of NAFLD-enhanced APAP hepatotoxicity remains unclear. In our previous experiment, we observed the effects of different doses of APAP on normal mice. The results showed that 100 mg/kg APAP could slightly promote lipid accumulation in normal mice (not shown). Meanwhile, some investigators suggested that basal oxidative stress and lower GSH level in NAFLD (Michaut et al., 2014). Hence, it is essential to further explore therapeutic targets for APAP-aggravated hepatic injury in NAFLD.

Recently, oxidative stress reduction has been considered a potential therapeutic target in APAP toxicity (Masarone et al., 2018; Yan et al., 2018). Meanwhile, NAFLD is also usually accompanied by oxidative stress, which in turn promotes lipid accumulation in the liver (Borrelli et al., 2018). Growing evidence has also shown that oxidative stress contributes to hepatic histopathologic changes by promoting the formation of lipid peroxide and decreasing the levels of SOD and GSH (Borrelli et al., 2018; Cui et al., 2020). Therefore, in the present study, we used a high-fat diet animal model that is similar to the pathological manifestation of early stage NAFLD, meanwhile, the alcohol and oleic acid mixture and L02 cells were used to establish steatosis model *in vitro*.

We measured the body weight of mice every week and the final liver index. The results showed that the mice lost weight after CSD treatment, and liver index elevation caused by HFD and APAP were significantly decreased (Figure 3A,B). In addition, APAP significantly increased the activity of serum ALT and AST and caused further hepatic structural damage and increase of fat vacuoles. SREBP-1c, a key regulator of liver lipid metabolism, can promote the synthesis of liver triglycerides and fatty acids (Li et al., 2018), therefore, the analysis of TG and SREBP-1c protein as well as oil red staining suggested increased lipid accumulation. These results were consistent with a previous study showing that APAP aggravated fat accumulation in mice with NAFLD (Shi et al., 2019). Moreover, APAP increased the levels of ROS and MDA, which are regarded as biomarkers for oxidative injury. Additionally, it led to a reduction in the antioxidant effect of SOD. Interestingly, we found that the expression of CAV1 protein was decreased in the process. Furthermore, after the use of NAC, the levels of the oxidative stress of HFD + APAP mice was ameliorated, and HE and oil red O staining and TG assay results showed that fat accumulation in the mice liver in HFD + APAP group was significantly reduced after NAC treatment.

CAV1, a regulator of redox homeostasis, can be modulated by reactive species in its expression, degradation, membrane trafficking, and posttranslational modifications, whereas the reactive species can be scavenged by CAV1-targeted treatments (Martinez-Outschoorn et al., 2015; Shao et al., 2020). It has been found to play a protective role in NAFLD in our previous work (Xue et al., 2020). CSD peptide is a stable analog of the active part of CAV1, which can be utilized for *in vivo* and *in vitro* studies. In the current study, repletion of CAV1 function through intraperitoneal administration of the CSD peptide resulted in amelioration of APAP-induced fat accumulation in mice with NAFLD.

*In vitro*, this was confirmed by the analysis of overexpression or silencing of CAV1 which showed that CAV1 could reduce ROS elevation in A/O-induced L02 cells stimulated by APAP, thus reducing lipid accumulation. These results elucidate the protective effect of CAV1 on APAP-aggravated liver injury in NAFLD. Although a study suggested that APAP-induced reduced hepatotoxicity in CAV1 KO animals was not related to alterations in the antioxidant defense (Gardner et al., 2010). Different from that study, in current study, APAP was used for animals in NAFLD state. As previously mentioned, NAFLD increases the vulnerability of the liver to many factors. Furthermore, a study reported that antioxidants benefit people with elevated levels of oxidative stress more than people with low levels of ROS, because ROS also play a critical physiological role in cell homeostasis (Senoner and Dichtl, 2019). Therefore, the role of antioxidant molecules may be different in different pathological states. Interestingly, we found that CAV1 alleviates oxidative stress in the liver of APAP-treated mice with NAFLD. However, the specific mechanism for this beneficial effect of CAV1 is poorly understood.

Recently, AMPK, a diffusely known energy sensor, has been reported to play a crucial role in antioxidant responses (Wu et al., 2018; Lin et al., 2020). We measured AMPK activity by detecting phosphorylation of AMPKα subunit at threonine 172. The data showed that AMPK activity was impaired in A/O-stimulated L02 cells and that it was further impaired after APAP administration, which was also consistent with the results *in vivo*. Nrf2, a key regulator of cells redox homeostasis, binds to Kelch-like ECH-associated protein 1 (Keap-1) in the cytoplasm (Xu et al., 2018). Upon stimulation, Nrf2 dissociates from the complex and subsequently enters the nucleus to promote the expression of antioxidant proteins such as HO-1 (Xu et al., 2018; Feng et al., 2019). AMPK activation is reported to facilitate the nuclear expression of Nrf2 through its downstream signaling to alleviate cellular oxidative stress (Wu et al., 2018). In addition, several reports have shown that liver protection can be achieved by activating the AMPK/Nrf2/HO-1 pathway (Shen et al., 2019; Kim et al., 2020). Notably, it has been shown that CAV1 regulates AMPK activation (Salani et al., 2012; Liu et al., 2015). Our results showed that the activity of AMPK was upregulated in CSD-treated mice. The protein levels of HO-1 and Nrf2 in the nucleus were also increased whereas Nrf2 level in the cytoplasm was reduced accordingly. These findings indicate that CAV1 mediated the activation of AMPK and consequently increased nuclear expression of Nrf2.

Similarly, *in vitro* experiments revealed that overexpressed CAV1 upregulated the expression level of p-AMPK and Nrf2 nuclear expression, and increased HO-1 level. In contrast, when CAV1 expression was suppressed by transfected CAV1-siRNA, p-AMPK, nuclear Nrf2, and HO-1 were markedly downregulated, and Oil Red staining demonstrated more extensive lipid droplets. These results suggest that CAV1 might be related to the AMPK/Nrf2/HO-1 pathway in alleviating liver injury induced by APAP. In addition, Nrf2 nuclear expression and HO-1 expression in the HFD and APAP groups were significantly higher than those in the NC group, which may be a crucial compensatory mechanism for the body to fight against oxidative stress. Pre-incubating cells with the AMPK inhibitor compound C inhibited AMPK activation induced by overexpression of CAV1 and reduced its ability to alleviate oxidative stress and lipid deposition.

Notably, we found that the protein expression of CAV1 was significantly suppressed after the use of compound C. It was observed that, the inhibition of AMPK activity led to increased lipid accumulation. Interestingly, it was proposed that the loss of AMPK activity does not affect hepatic fat accumulation, but it is a physiological correlation under normal conditions (Zhao and Saltiel, 2020). The impairment of liver AMPK activity has been proposed to be a key pathological event in the development of metabolic disorders related to metabolic syndrome (including liver steatosis) (Boudaba et al., 2018). Inhibition of AMPK can stimulate anabolic pathways, such as lipid synthesis, and attenuate catabolic pathways, such as β-oxidation (Boudaba et al., 2018). In addition, liver ROCK1 activation caused by obesity was reported to inhibit AMPK activity, leading to the increase of SREBP-1c mediated lipogenesis pathway, thus driving liver lipid accumulation (Huang et al., 2018). Our previous study also showed that inhibition of AMPK activation could promote lipid accumulation in NAFLD by inhibiting autophagy (Shi et al., 2019). It was reported that sterol regulatory element binding proteins (SREBPs) expression was mainly regulated by cellular cholesterol and CAV1 was negatively regulated by SREBPs (Xu et al., 2010; Fernandez-Rojo and Ramm, 2016). Besides, several studies have suggested that there is a molecular loop of reciprocal control between liver CAV1 and lipid metabolism (Gong et al., 2014; Fernandez-Rojo and Ramm, 2016). Therefore, the decreased CAV1 level after inhibiting AMPK activation may be the consequence of abnormal lipid metabolism and increased SREBPs protein expression in cells. In addition, the protein expression of CAV1 was reported to be possibly regulated by AMPK/eNOS/NF-κB/Sp1 circuit loop (Bai et al., 2017). However, it remains unclear how CAV1 and AMPK interact. A study has indicated that CAV1 may affect the activation of AMPK by regulating energy balance (Salani et al., 2012), which requires elucidation.

## Conclusion

**FIGURE 10:**
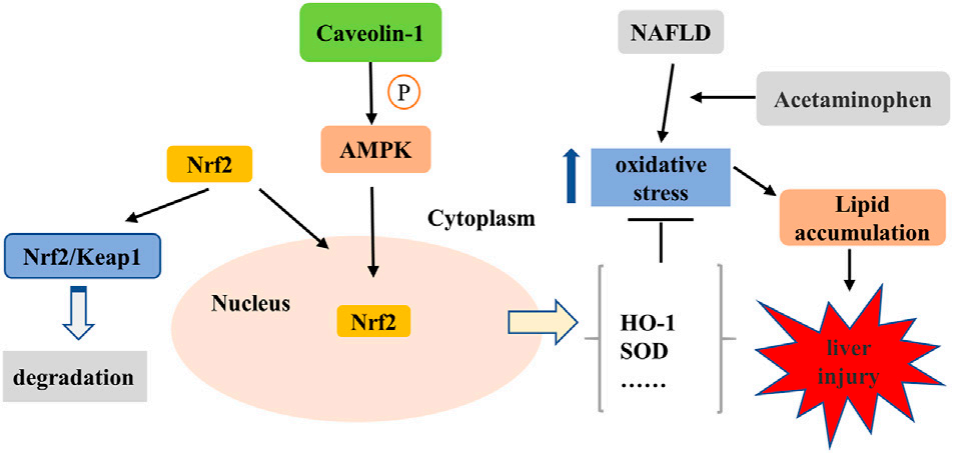
CAV1 attenuated APAP-induced liver injury in NAFLD associated with suppressing oxidative stress by activating AMPK/Nrf2/HO-1 pathway. APAP exacerbated oxidative stress in NAFLD, leading to increased lipid deposition and liver damage. It is known that Nrf2 binds to Kelch-like ECH-associated protein 1 (Keap-1) and locates in the cytoplasm under physiological conditions. The activation of AMPK mediated by CAV1 could promote Nrf2 to dissociate from the complex and subsequently enters to the nucleus to enhance the expression of antioxidant enzymes in the cell. Thus, CAV1 attenuated APAP-induced liver injury in NAFLD associated with oxidative stress and fat accumulation by activating AMPK/Nrf2/HO-1 pathway.

In conclusion, the current study revealed that CAV1 notably promoted the nuclear expression of Nrf2 *via* the AMPK pathway, which ultimately resulted in increased expression of the anti-oxidative protein and alleviated oxidative stress and lipid deposition in both mice and L02 cells (Figure 10). The results after NAC treatment show that inhibiting oxidative stress is beneficial to reducing lipid deposition. However, the effects of CAV1 diminished and oxidative stress increased when AMPK was suppressed, which supports the possibility that CAV1 may inhibit oxidative stress through AMPK/Nrf2/HO-1 pathway, thereby alleviating APAP-induced lipid deposition in NAFLD. Thus, CAV1 might be a therapeutic target of APAP-aggravated hepatotoxicity in NAFLD.

## Data Availability

The original contributions presented in the study are included in the article/supplementary materials, further inquiries can be directed to the corresponding author.
